# Miss Marguerite Washington

**Published:** 1917-02

**Authors:** 


**Miss Marguerite Washington,** a Philadelphia painter of portraits and miniatures and widely known to the medical profession for her anatomic illustrations, died Jan. 11. She was a great great-grand-niece of President George Washington.

